# Seed Yield of Mungbean (*Vigna radiata* (L.) Wilczek) in relation to Growth and Developmental Aspects

**DOI:** 10.1100/2012/425168

**Published:** 2012-08-02

**Authors:** M. M. A. Mondal, A. B. Puteh, M. A. Malek, M. R. Ismail, M. Y. Rafii, M. A. Latif

**Affiliations:** ^1^Department of Crop Science, Faculty of Agriculture, Universiti Putra Malaysia (UPM), 43400 Serdang, Selangor, Malaysia; ^2^Crop Physiology Division, Bangladesh Institute of Nuclear Agriculture, Mymensingh 2202, Bangladesh; ^3^Plant Breeding Division, Bangladesh Institute of Nuclear Agriculture, Mymensingh 2202, Bangladesh; ^4^Institute of Tropical Agriculture, Universiti Putra Malaysia (UPM), 43400 Serdang, Selangor, Malaysia; ^5^Plant Pathology Division, Bangladesh Rice Research Institute, Joydebpur, Gazipur, Bangladesh

## Abstract

Growth parameters such as leaf area (LA), total dry mass (TDM) production, crop growth rate (CGR), relative growth rate (RGR), and net assimilation rate (NAR) were compared in six varieties of mungbean under subtropical condition (24°8′ N 90°0′ E) to identify limiting growth characters for the efficient application of physiology breeding for higher yields. Results revealed that a relatively smaller portion of TDM was produced before flower initiation and the bulk of it after anthesis. The maximum CGR was observed during pod filling stage in all the varieties due to maximum leaf area (LA) development at this stage. Two plant characters such as LA and CGR contributed to the higher TDM production. Results indicated that high yielding mungbean varieties should possess larger LA, higher TDM production ability, superior CGR at all growth stages, and high relative growth rate and net assimilation rate at vegetative stage which would result in superior yield components.

## 1. Introduction

Mungbean is one of the most important pulse crops for protein supplement in subtropical zones of the world. It is widely grown in Indian subcontinent as a short duration catch crop between two principal crops. Mungbean contains 51% carbohydrate, 24–26% protein, 4% mineral, and 3% vitamins [1]. Besides providing protein in the diet, mungbean has the remarkable quality of helping the symbiotic root rhizobia to fix atmospheric nitrogen and hence to enrich soil fertility [2].

In spite of the best efforts for improving the mungbean varieties, the yield of this crop remains low. Several studies have been made to understand their performances which mainly include the contribution of various yield components towards yield [3–6]. The yield components depend on some physiological traits. To understand the physiological basis of yield difference among the genotypes of mungbean, it is essential to quantify the components of growth, and the variation, if any, may be utilized in crop improvement. Variation in dry matter accumulation and pod production in different genotypes may be related to some factors such as leaf area (LA), crop growth rate (CGR), net assimilation rate (NAR), and relative growth rate (RGR). Pandey et al. [7] analyzed growth parameters of five varieties of black gram in order to study the physiological causes of yield differences and observed the differences in CGR, NAR, RGR, and LA among the varieties. Egli and Zhen-wen [8] suggested that seeds per unit area were related to canopy photosynthesis during flowering and pod set and canopy photosynthesis rate was determined through LAI and CGR. A plant with optimum LAI and NAR may produce higher biological yield as well as seed yield [9]. The dry matter accumulation may be the highest if LAI attains its maximum value within the shortest possible time [4, 10, 11]. Not only TDM production, but also the capacity of efficient partitioning between the vegetative and reproductive parts may produce high economic yield [12, 13]. Probably a better understanding of crop growth and yield parameters and the partitioning of assimilates into seed formation would help to expedite yield improvement of this crop. It was with this aim that the present investigation was carried out.

## 2. Materials and Methods

The experiments were carried out at the experimental field of Bangladesh Institute of Nuclear Agriculture (BINA), Mymensingh (24°8′ N 90°0′ E), Bangladesh in Kharif-I (February-May) season of 2010 and 2011. Six mungbean varieties of which three high (BARImung-4, BINAmung-7, and BUmung-1) and three low (BARImung-6, BINAmung-6, and BUmung-2) yielding varieties were used as planting material. The soil of the experimental area is silty loam having a total of 0.065% nitrogen, 1.17% organic matter, 18.5 ppm available phosphorus, 0.30 meq/100g exchangeable potassium, 20 ppm sulphur and 6.8 pH. The experiments were laid out in a randomized complete block design with three replicates in both the years. Urea, triple superphosphate, muriate of potash, and gypsum were used as a source of nitrogen, phosphorus, potassium, and sulphur at the rate of 40, 120, 80, and 30 kg ha^−1^, respectively at the time of final land preparation. Seeds were sown on 10 and 14 March for the year of 2010 and 2011, respectively. A unit plot size of 20 m^2^ (5 m × 4 m) with plant spacing of 30 cm × 10 cm was used. Cultural practices were the same in both the seasons. Seeds were sown in line, and two weeks after germination, the plants were thinned to a density of 30 plants m^−2^. First weeding was done followed by thinning at about 20 days after sowing (DAS). A single irrigation was given at 21 DAS at both years. Insecticide (Ripcord 50 EC at 0.025%) was sprayed at flowering and fruiting stage (45 and 55 DAS) to control shoot and fruit borer. 

To study ontogenetic growth characteristics, a total of five harvests were taken in both years. The second and third rows of each plot were used for sampling. The first crop sampling was done at 25 DAS, and continued at an interval of ten days up to 65 DAS, that is, till attaining physiological maturity. From each sampling, a unit area of 0.3 m^2^ in two adjacent rows of 0.5 m (10 plants) was randomly selected from each plot and uprooted for collecting necessary parameters. The plants were separated into roots, stems, leaves, and pods, and the corresponding dry weight was recorded after oven-drying at 80 ± 2°C for 72 hours. The leaf area of each sample was measured by automatic leaf area meter (Model: LICOR 3000, USA). The growth analyses like crop growth rate (CGR), relative growth rate (RGR), and net assimilation rate (NAR) were carried out following the formulae of Hunt [14]. The number of opened flowers plant^−1^ was recorded from 15 randomly selected pants, that is, five from each plot. The opened flowers were counted daily. Reproductive efficiency (per cent pod set to opened flowers) was calculated as follows: (number of pods plant^−1^ ÷ number of opened flowers plant^−1^) × 100. The yield contributing characters were recorded at harvest from ten competitive plants of each plot. The seed yield was recorded from five rows of each plot (1.50 m × 3.0 m) and converted into seed weight plant^−1^ by dividing the plant number. Harvest index was calculated from the collected data using formula: (economic yield plot^−1^ ÷ biological yield plot^−1^) × 100. 

Data were analyzed statistically as per the design used following the analysis of variance (ANOVA) technique and the mean differences were adjusted with DMRT at 5% level of significance using the statistical computer package programme, MSTAT-C following Russell [15]. 

## 3. Results and Discussion

### 3.1. Growth Parameters

**Figure 1:**
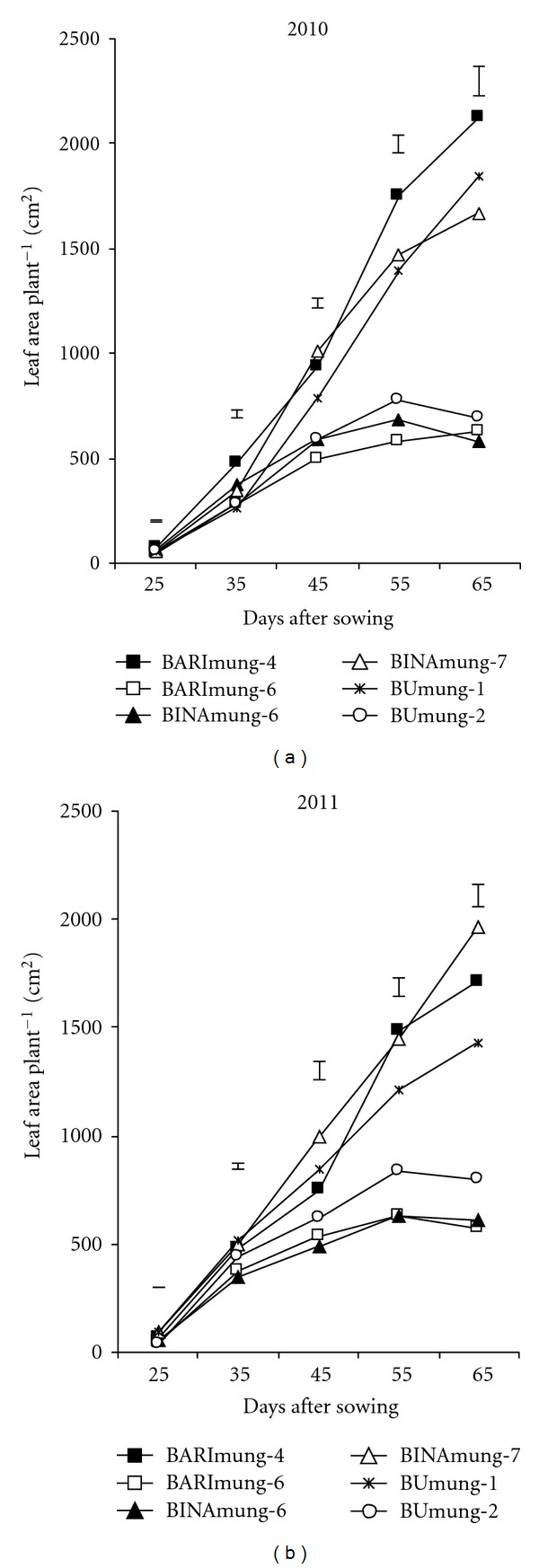
Leaf area development at different ages in six mungbean varieties. Vertical bars represent LSD (0.05).

**Figure 2:**
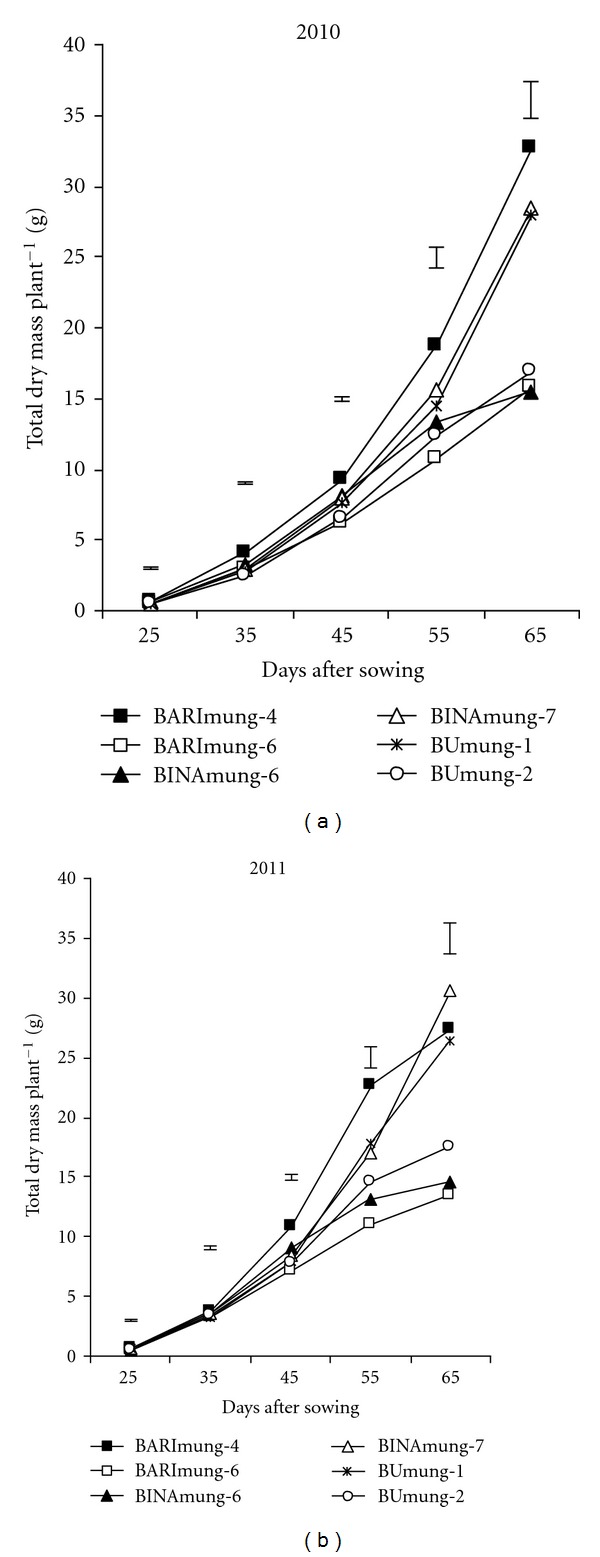
Changes in total dry mass at different ages in six mungbean varieties. Vertical bars represent LSD (0.05).

The differences among the varieties for leaf area (LA) and total dry mass (TDM) were significantly different at all growth stages (Figures 1 and 2). The differential varietal performance for LA and their relation to the DM production, at each growth stage, could be associated to the genetic makeup of the varieties. A common feature of mungbean genotypes was slow in TDM accumulation and LA development during the first 35 DAS followed by a rapid increase after commencement of flowering. It is mentionable that flowering was started at 40–45 DAS, depending on variety. The faster TDM accumulation after the starting of reproductive stage was the result of increased LA [11]. The TDM production in all genotypes increased with age till physiological maturity whereas in LA followed a typical sigmoid pattern in three varieties with respect to time. The LA in both years increased till 65 DAS (pod development stage) in three varieties (BARImung-4, BINAmung-7, and BUmung-1) out of six whereas in the rest three varieties (BARImung-6, BINAmung-6, and BUmung-2), the LA increased up to 55 DAS followed by a decline because of abscission of old leaves. It is mentionable that the early three varieties (BARImung-4, BINAmung-7, and BUmung-1) matured within 72–75 DAS, and latter three varieties (BARImung-6, BINAmung-6 and BUmung-2) matured within 60–65 DAS (Table 1). So, at 65 DAS, the latter three varieties were at mature stage whereas the earlier three varieties were then at pod development stage. Therefore, the LA was declining in trend in latter three varieties at 65 DAS for leaf shedding. Results indicated that high yielding genotypes (BARImung-4, BINAmung-7, and BUmung-1) always showed superiority in TDM and LA production compared to low yielding ones (BARImung-6, BINAmung-6, and BUmung-2) at most of the growth stages. These results indicate that LA and TDM are the most important parameters for increasing seed yield in mungbean. This result is consistent to Ahmed et al. [16] who observed that seed yield depends on LA and TDM production in mungbean. 

**Figure 3:**
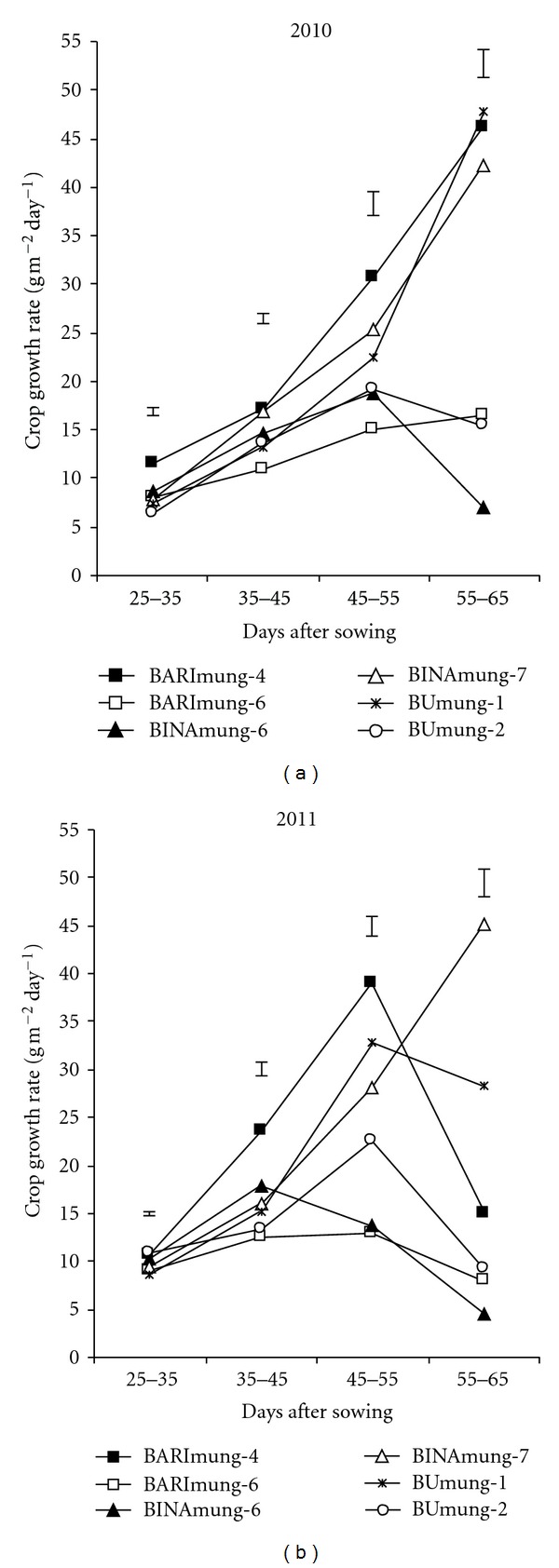
Pattern of crop growth rate in six mungbean varieties during their growth period. Vertical bars represent LSD (0.05).

The crop growth rate (CGR) in four varieties (BARImung-4, BARImung-6, BINAmung-7, and BUmung-1) tended to increase with the advancement of stage till 55–65 DAS in 2010 and similar trend was observed in one variety of BINAmung-7 in 2011 (Figure 3). The other varieties in both the years showed a typical sigmoid pattern with respect to time. Varieties differed significantly in CGR at all growth stages in the both years. Results indicated that high yielding varieties (BARImung-4, BINAmung-7, and BUmung-1) showed superiority in CGR over three low yielding varieties (BARImung-6, BINAmung-6, and BUmung-2) at most of the growth stages. It is evident that high yielding variety had two distinct growth phases: early slow growth (up to 35 DAS, before flowering start), followed by a rapid growth (35–65 DAS, flowering and pod filling stage). The low yielding varieties had steady growth rate up to 55 DAS followed by a decline. The slow growth rate at early growth stage was associated with lower LA and TDM production. The initial slow growth favours weed growth and development; thus crop ultimately suffers a loss. The selection of genotypes with rapid growth rate in early part of a crop life is therefore warranted. In the present experiment, high yielding varieties (BARImung-4, BINAmung-7, and BUmung-1) had greater CGR than low yielding ones (BARImung-5, BINAmung-6, and BUmung-2), that is, the desirable character. At the later stage of pod growth and development (55–65 DAS), there was a decline in CGR, of three low yielding varieties possibly owing to similar decline in LA during this stage (Figure 1).

**Figure 4:**
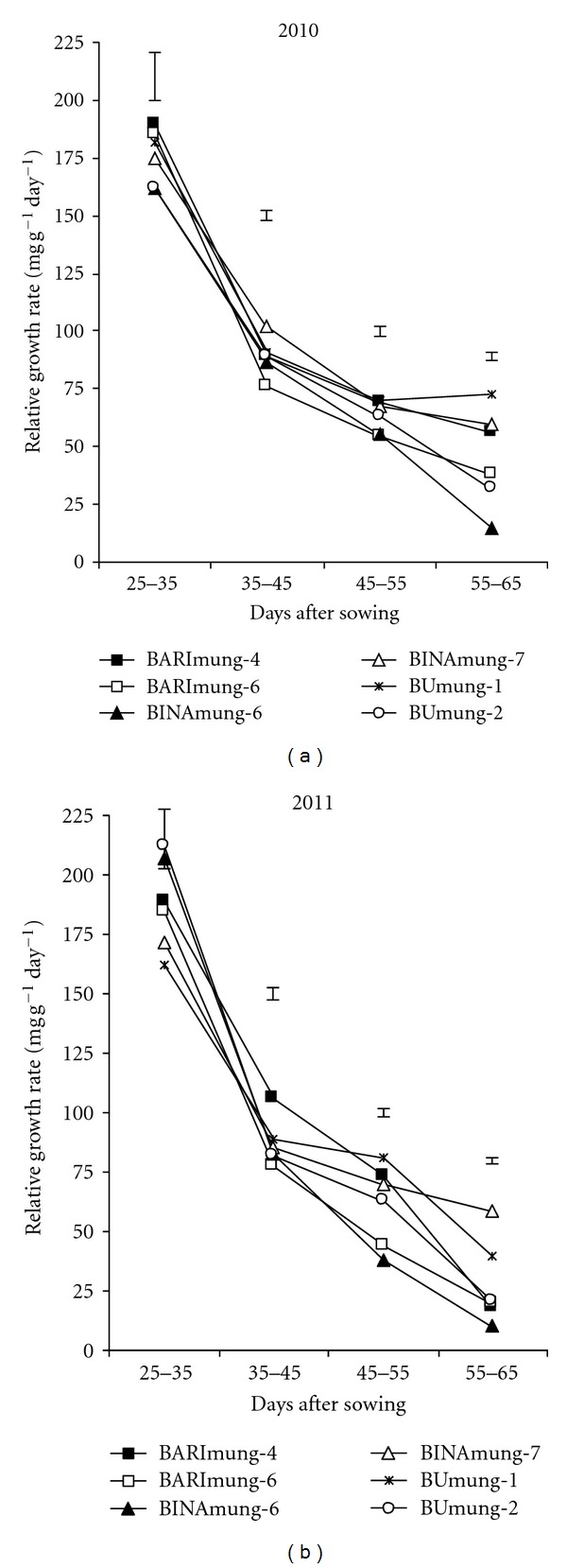
Pattern of relative growth rate in six mungbean varieties during their growth period. Vertical bars represent LSD (0.05).

The relative growth rate (RGR) declined with age in both years (Figure 4). In 2010, the RGR was higher in high yielding genotypes than low yielding ones at early growth stages whereas, in 2011, it has not shown the similar trend. In 2011, the higher RGR was recorded in BUmung-2 and BINAmung-6, the low yielding varieties. Kollar et al. [17] observed a decrease in RGR as the season advanced.

**Figure 5:**
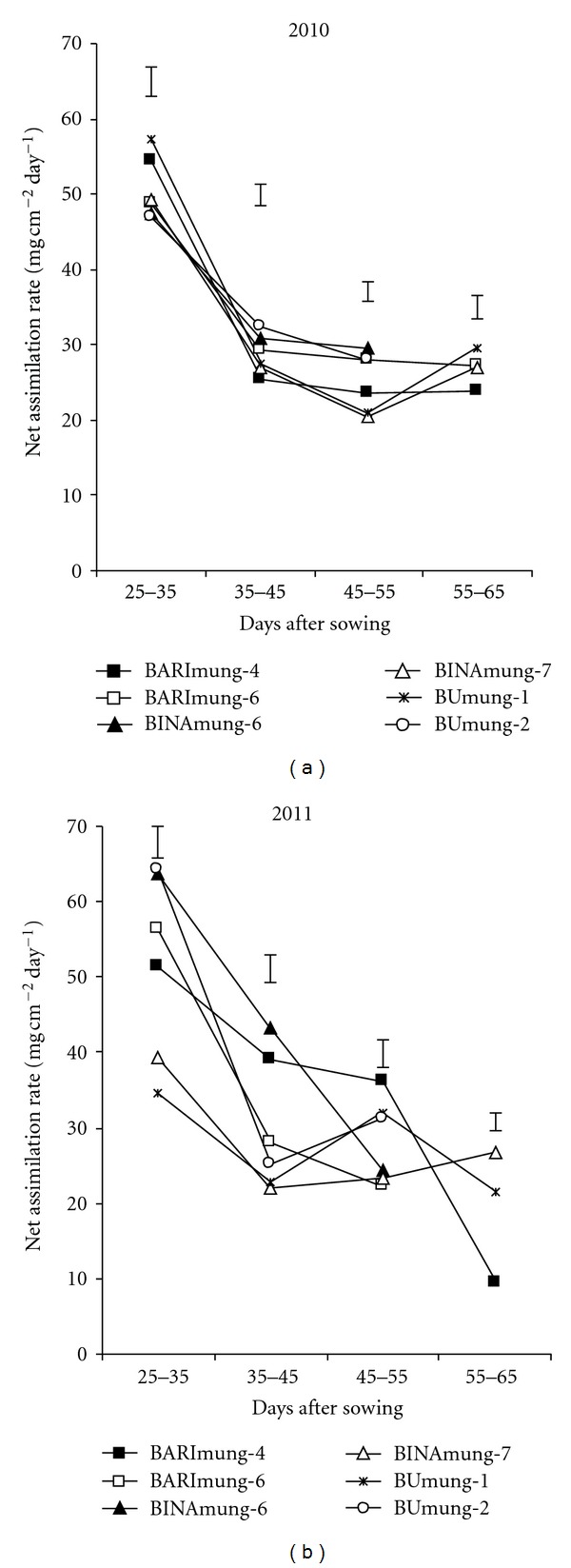
Showing net assimilation rate of four mungbean genotypes at different ages. Vertical bars represent LSD (0.05).

The pattern of net assimilation rate (NAR) in both seasons was not similar (Figure 5). The changes in NAR varied widely in two seasons at different times and were brought about through plastic changes in leaves which in turn were induced by the light environment of the growth season. The NAR was rapidly declined with age in both years till 35–45 DAS followed by being slowly declined in some varieties. In 2010, the NAR increased in BINAmung-7 and BUmung-1 from 45–55 DAS to 55–65 DAS. Similar trend was also observed in 2011 for BINAmung-7. In 2010, at 25–35 DAS, the highest NAR was recorded in BUmung-1 and BINAmung-4, the high yielding varieties whereas, in 2011, the higher NAR was observed in BUmung-2 and BINAmung-6, the low yielding genotypes. These results indicate that the NAR did not follow any definite pattern among the genotypes over years. In both years, the lower NAR was observed in both high and low yielding varieties indicating NAR had no relation with yield in mungbean. The rise in NAR of some varieties during reproductive stage was probably due to increased demand of assimilation by the growing seed fraction [18]. However, the NAR in the both seasons was maximal between 25 and 35 DAS, at vegetative stage. The NAR declined at later growth stages (reproductive stage) which may be attributed to excessive mutual shading as the LA was maximum during this period and increased number of old leaves could have lowered the photosynthetic efficiency [19]. In grain legume, the excess LA was reported to have lowered NAR drastically and resulted in a decreased dry matter accumulation, which probably resulted from excessive mutual shading [7]. 

### 3.2. Morphological, Reproductive, and Phenological Parameters 

The morphological (plant height and branch number plant^−1^), phenological (flowering duration and days to maturity), and reproductive characters (number of opened flowers plant^−1^ and reproductive efficiency) differed significantly among the studied cultivars (Table 1). The effect of year had no significant different in most of the plant characters. Therefore, mean values over two years are presented here. Results showed that high yielding varieties (BARImung-4, BINAmung-7, and BUmung-1) had taller plants (range 56.6–60.3 cm) with higher number of branches (range 2.29–2.77 plant^−1^) than the low yielding ones (range 38.2–40.3 cm for plant height and 0.69–0.72 plant^−1^ for branch number). The flowering duration, number of opened flowers plant^−1^, and days to maturity were also higher in high yielding varieties than the low yielding ones but the reverse trend was observed in case of reproductive efficiency. It seems that flowering duration and number of open flower have positive relation with seed yield in mungbean. The present result is consistent with Mondal et al. [20] who reported that flowering duration and flower production had relation with seed yield in mungbean.

### 3.3. Yield Components and Seed Yield 

The three varieties, BARImung-4, BINAmung-7, and BUmung-1, produced higher grain yield than the other three varieties, BARImung-6, BINAmung-6, and BUmung-2, due to production of higher number of pods plant^−1^ (Table 2). Although the low yielding varieties, BARImung-6, BINAmung-6, and BUmung-2, produced larger pod and bolder seed size with superior harvest index than the other three high yielding varieties (BARImung-4, BINAmung-7, and BUmung-1), yet they showed lower yield due to fewer number of pod production. Mondal et al. [13] observed that seed yield of mungbean had no positive relation with pod and seed size as well as harvest index. In present experiment, similar result was also observed. Results further revealed that genotypes which had higher LA, TDM, and CGR also produced higher seed yield in mungbean. 

From the results, it appeared that, for getting superior characters of yield components, a high yielding mungbean genotype should posses a relatively larger leaf area with superior growth parameters.

## Figures and Tables

**Table 1:** Variation in morphological, reproductive, and phenological parameters in six mungbean varieties (mean over two years).

Variety	Plant height (cm)	Branches plant^−1^ (no.)	Flowering duration (days)	Total opened flowers plant^−1^ (no.)	Reproductive efficiency (%)	Days to maturity
BARImung-4	60.3 a	2.77 a	32.5 a	80.3 a	44.9 c	74.7 a
BARImung-6	38.2 c	0.69 c	10.3 c	19.2 d	80.0 a	60.0 d
BINAmung-6	40.3 c	0.72 c	10.2 c	19.0 d	79.3 a	60.9 d
BINAmung-7	56.6 b	2.66 a	33.5 a	72.7 b	56.0 b	73.7 a
BUmung-1	58.1 ab	2.29 b	25.2 b	55.2 c	57.2 b	71.5 b
BUmung-2	38.8 c	0.70 c	11.1 c	18.9 d	81.8 a	64.5 c

*F*-test	∗∗	∗∗	∗∗	∗∗	∗∗	∗∗

CV (%)	5.09	6.76	7.19	7.29	5.19	2.83

In a column, figures bearing the same letter(s) do not differ significantly at *p* ≤ 0.05 by DMRT; ^∗∗^ indicates significance at 1% level of probability.

**Table 2:** Yield components and seed yield in six mungbean varieties (Mean over two years).

Variety	Pods plant^−1^ (no.)	Pod length (cm)	Seeds pod^−1^ (no.)	1000-seed weight (g)	Seed yield plant^−1^ (g)	Harvest index (%)
BARImung-4	36.0 b	6.98 c	10.30	33.4 c	10.82 b	26.65 b
BARImung-6	15.4 d	8.74 a	10.45	53.9 a	7.46 d	33.94 a
BINAmung-6	15.1 d	8.68 ab	10.40	51.7 a	7.03 de	31.81 a
BINAmung-7	40.8 a	6.53 d	10.15	32.1 c	11.57 a	28.16 b
BUmung-1	31.6 c	7.17 c	10.40	34.0 c	9.68 c	26.27 b
BUmung-2	15.3 d	8.42 b	10.40	49.2 b	6.78 e	28.51 b

*F*-test	^ ∗∗^	^ ∗∗^	NS	^ ∗∗^	^ ∗∗^	^ ∗∗^

CV (%)	8.41	3.30	3.46	5.20	4.04	7.01

In a column, figures bearing the same letter(s) do not differ significantly at *p* ≤ 0.05 by DMRT; NS indicates not significant; ^∗∗^ indicates significance at 1% level of probability.
